# Clinical severity of pulmonary Tuberculosis (TB) is associated with Hypocholesterolemia among adult patients in Uganda

**DOI:** 10.4314/ahs.v26i2.2

**Published:** 2026-06

**Authors:** Edirisa Kibuuka, John Mukisa, Ezekiel Mupere

**Affiliations:** 1 Child and family foundation, Uganda; 2 Makerere University School of Health Sciences, Clinical Epidemiology Unit; 3 Makerere University, college of health sciences, School of Biomedical Sciences, Department of Immunology & Molecular Biology; 4 College of Health Sciences Makerere University, Paediatrics & Child Health; Clinical Epidemiology Unit

**Keywords:** Tuberculosis, Wasting, Hypocholesterolemia

## Abstract

**Background:**

Hypocholesterolaemia in pulmonary tuberculosis(TB) patients is disregarded but is a predictor of poor survival.

**Objectives:**

To determine serum lipid profile differences at diagnosis and various times of medication based on sex, HIV(human immune virus) and wasting status and factors associated with hypocholesterolaemia.

**Methods:**

A repeated cross-sectional study was conducted to assess triglyceride(TG), total cholesterol (TC), high-density lipoprotein(HDL) and low-density lipoprotein(LDL) among 414 participants.

**Results:**

Majority of the participants were males(239/414)(58%), with a median age of 28years. TB patients showed significantly lower lipid profile at diagnosis regardless of HIV-status and sex. Presence of anaemia among HIV-seropositive participants with TB, prevalence ratio(PR):2.6, 95%confidence interval(CI):1.0-7.1, p<0.05) and HIV-seronegative participants with TB(PR:2.5, 95%CI:1.1-5.5, p<0.05), moderate/severe TB(PR:2.5,95%CI:1.1-5.5, p<0.05) and CD4 cells>200(PR:0.3 95%CI:0.1-0.8, p<0.05) among HIV-seropositive participants with TB were associated with HDL-hypocholesterolaemia. Male sex, moderate/severe TB, body wasting, dyspnoea and chest pain were independently associated with TC-hypocholesterolaemia. Regardless of sex and wasting status, the pattern of cholesterol recovery was similar at varying times

**Conclusion:**

We envisage that hypocholesterolemia is a marker of clinical TB-disease severity and its recovery indicates treatment response.

## Introduction

TB incidence and mortality are anticipated to increase by 15% from 10million new cases and 1.5 million deaths globally^1^. About 2.4million new cases and 665,000 deaths are reported in sub-Saharan Africa while in Uganda, 196 and 16/100,000 cases occur respectively^2^, ^3^. TB related morbidity and mortality is associated with dyslipidemia^4^. In Africa, the prevalence of hypocholesterolemia is 28.5% with no sex differences, common among thin rural dwellers and those with poor nutritional-status^5^-^7^.

Hypocholesterolemia a consequence of body wasting and inflammatory responses suppresses leptin production and oxidative/antioxidation equilibrium^8^. These processes affect cell mediated immunity and lung function leading to severe disease, delayed sputum conversion and mortality^9^, ^10^.

In Uganda, a low treatment success among HIV-seronegative and coinfected individuals is due to unsatisfactory monitoring of TB treatment response^2^, ^11^. Weight gain, chest X-rays, sputum microscopy and culture used in monitoring treatment response have shortcomings^12^, ^13^. GeneXpert is highly sensitive but only used for TB diagnosis^14^. World health organization (WHO), National Tuberculosis and Leprosy Programs(NTLP) and Ministry of Health(MoH) Uganda recommend bacteriological, clinical and nutritional assessments for monitoring treatment response^15^, ^16^. However, when performed, nutritional assessment stops at taking anthropometry and yet is a precise marker of treatment response.

Lipid profile recovery in TB patients indicates sputum sterility, immune function recovery and treatment adherence^17^, ^18^. In this study, we ascertained lipid profile differences among patients with TB/HIV and individuals without disease at diagnosis. We identified factors associated with hypocholesterolemia; investigated lipid profile at various times of treatment based on sex, wasting status and HIV-serostatus. The study aimed at determining whether clinical TB-severity among coinfected and TB patients is associated with hypocholesterolemia.

## Materials and methods

### Design and setting

We conducted a repeated cross-sectional study among 414 adult TB patients at Mulago Hospital using secondary data. Participants were consecutively enrolled at diagnosis (0), months 2, 5 and 6/8. HIV patients were recruited from infectious disease institute located 500meters from the NTLP clinic. Individuals without disease were recruited from the community where patients resided. TB patients were initiated on treatment and monitored with sputum microscopy and culture^15^.

### Measurements

Structured and pretested questionnaires were used to obtain patients' information and clinical characteristics. At enrollment, participants underwent physical examination, chest radiography, GeneXpert/sputum microscopy/culture to confirm TB. A COBAS 6000 (Roche Diagnostics Ltd), SYSMEX XE-2100 and SOFT STYLE® glucometer analyzed lipid profile, full blood hemogram and fasting blood glucose respectively. All participants underwent HIV-testing following the national HIV serial testing algorithm^19^.

Nutritional status was assessed using height and weight measured with a SECA-digital electronic scale and stadiometer. Body mass index (BMI) was calculated as weight in kilograms divided by height in square-meters. Mid upper arm circumference (MUAC) was performed on the arm using non-stretchable measuring tape. Bioelectric impedance analysis(BIA) was performed to determine resistance and reactance to measure fat and fat-free mass using equations that were cross-validated^20^. Equipment was calibrated using the manufacturer's guide before performing any measurements

Clinical TB-severity was measured using a TB-score consisting of 13 variables each scoring 1 point as follows^21^. Self-reported symptoms of cough, hemoptysis, dyspnea, chest pain and night sweating; anemic conjunctivae, tachycardia, positive findings at lung auscultation, axillary temperature>37.00C, BMI (<18, <16), MUAC (<220mm, <200mm). Severity classes were collapsed in mild≤5 and moderate/severe>5.

### Eligibility criteria

We included adults with an initial episode of TB and residing in Kampala. All participants signed informed consent before enrollment. HIV-1 was confirmed using enzyme-linked immunosorbent assay for HIV antibodies. Diabetic patients (blood glucose≥126 mg/dl) and those taking statins and fibrates were excluded from the study.

### Operational definitions

HDL and TC-hypocholesterolemia were defined as <0.9 and <3.7mmol/l respectively^22^. BMI cutoffs of nonwasting and wasting were 18.5-24.5kg/m^2^ and ≤18.5kg/m^2^. Free-fat mass(FFMI) wasting among men <16.7kg/m^2^ and <14.6kg/m^2^ in women and fat mass(FMI)<1.8kg/m^2^ and <3.9kg/m^2^ respectively^20^.

### Sample size calculation

We estimated a sample size of 445 adults using Kish Leslie formular 1965, assuming 43.6% as prevalence rate, 0.05 precision level and considering 15% for missing data^23^, ^24^.

### Statistical analysis

Descriptive statistics were summarized as means, median, interquartile range and proportions. Means and proportions were compared using Wilcoxon-Mann-Whitney and Chi-square tests. Cox regression models with equal time assigned to all participants was used to estimate PR^25^-^27^. Clinical significance and a cut-off of p-value≤0.25 at bivariate were used to select variables for multivariable analysis^28^. Interaction was assessed with a chunk test and confounding at 10%. We assumed a power of 80%(1-β=0.80) and a type I error of 5%. All tests were two sided with a significant p-value≤0.05. Analyses were performed using STATA version 16.0. Participants with missing lipid profile results were excluded on analysis.

## Ethical consideration

Confidentiality was by concealment of patients' names hence use of identification numbers. The study was explained to participants before enrollment and written-informed consent obtained. Final approval was obtained from Makerere University School of Medicine Research and Ethics Committee and Uganda National Council of Science and Technology.

## Results

### Description of study population

**Fig 1:**
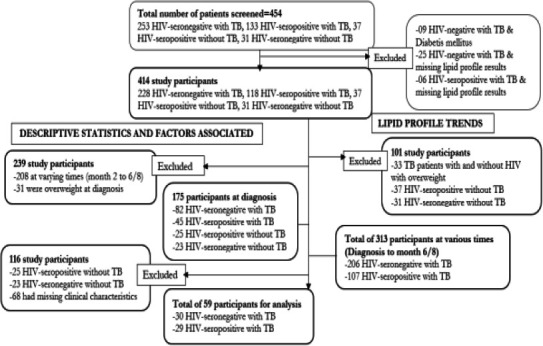
Profile of TB patients enrolled in the study at the National TB and Leprosy Program clinic, Mulago Hospital

Of the 454 participants, 40 were excluded because of diabetes mellitus and missing data leaving a total of 414 participants in the study(Figure 1).

Several participants were males (58%), with secondary education (45%), divorced (43%) and presented with mild TB (69%). Median age was 28years (Table 1).

**Table 1:** Participants characteristics and scheduled visits at Mulago Hospital

Characteristics(N)=414	N (%)
**Median age(years)interquartile range**	28(23-37)
**Sex**	
Female	175(42)
Male	239(58)
**Scheduled visits**	
Diagnosis	206(50)
Month 2	82(20)
Month 5	76(18)
Month 6/8	50(12)
**HIV-serostatus**	
Negative	259(63)
Positive	155(37)
**BMI(Kg/m^2^)**	
<18.5	111(27)
18.5-24.5	249(60)
>24.5	54(13)
LMI(Kg/m^2^)*	
Nonwasted	107(82)
Wasted	24(18)
FMI(Kg/m^2^)*	
Nonwasted	83(64)
Wasted	48(36)
Clinical TB-severity*	
Mild	90(69)
Moderate / Severe	41(31)
Marital status*	
Single	107(38)
Married	54(19)
Divorced	122(43)
Education level*	
Primary	117(41)
Secondary	126(45)
Tertiary	40(14)

* Missed data

Regardless of sex and HIV-serostatus, TB patients presented significantly lower lipid profile levels than participants without TB though TG was significantly higher (Table 2).

**Table 2:** Lipid profiles among TB patients at diagnosis attending Mulago Hospital

	Men(n=100)				Women(n=75)			

	HIV+(n=36)		HIV-(n=64)		HIV+(n=35)		HIV-(n=40)	
Lipid profiles (Mean±SD	TB+ (n=20)	TB- (n=16)	TB+ (n=49)	TB- (n=15)	TB+ (n=25)	TB- (n=10)	TB+ (n=32)	TB- (n=8)
**TC**	3.10±0.76	3.74±0.71^a^	3.47±0.94	4.16±0.90^a^	3.65±1.33	3.72±1.00	3.64±0.82	4.10±0.51
**HDL**	0.68±0.43	1.00±0.33^a^	0.86±0.50	1.12±0.23^a^	0.78±0.60	0.84±0.33	0.94±0.43	1.32±0.30^a^
**LDL**	1.66±0.50	2.23±0.65^a^	1.44±0.66	2.50±0.86^b^	1.95±0.90	2.35±0.80	1.59±0.72	2.31±0.38^a^
**TG**	1.57±0.88	1.13±0.36	1.70±0.97	1.10±0.62^a^	1.77±1.00	1.16±0.51^a^	1.80±1.00	0.92±0.44^a^

P-values obtained with Wilcoxon-Mannwhitney test

a p<0.05

b p<0.01

SD=Standard deviation, TB-=no Tuberculosis, TB+=Tuberculosis present, HIV+=HIV-seropositive, HIV-=HIV-seronegative

Anemia, moderate/severe TB, BMI, HIV and CD4>-200cells were associated with HDL-hypocholesterolemia at univariate. HIV and BMI confounded CD4-status for HDL-hypocholesterolemia. On stratification, HIV patients with CD4>200cells were 30% less likely to have HDL-hypocholesterolemia than those with CD4≤-200cells. Regardless of HIV-serostatus, participants with anemia were 3folds at risk of HDL-hypocholesterolemia than individuals without anemia. Moderate/severe TB among HIV patients increased risk of HDL-hypocholesterolemia by 3folds. Nonwasted patients with CD>-200cells were 10% less likely for HDL-hypocholesterolemia than those with CD4≤200cells (Table 3).

**Table 3:** Factors associated with HDL-hypocholesterolemia among TB patients at diagnosis

Characteristic						Stratified models			
	
						HIV+(n=29)		HIV-(n=30)	
		Univariate		Overall	model				
Overall(N)=59									
	N (%)	PR	95%CI	PR	95%CI	PR	95%CI	PR	95%CI
**Sex**									
Female	31(52)	1							
Males	28(48)	1.0	0.6-1.7						
**Age**									
=30	43(73)	1							
>30	16(27)	0.8	0.5-1.3						
**CD4cells**									
=200	20(34)	1		1		1		1	
>200	39(66)	0.4^b^	0.2-0.7	0.4^a^	0.2-1.0	0.3^a^	0.1-0.8	1	-
**HIV-serostatus**									
Negative	30(51)	1		1					
Positive	29(49)	2.4^b^	1.4-4.1	1.8	0.9-3.7				
**Chest pain**									
Absent	21(36)	1							
Present	38(64)	1.0	0.6-1.7						
**Hemoptysis**									
Absent	49(83)	1							
Present	10(17)	0.8	0.4-1.5						
**Anemia Hb,**									
**g/dl**									
>10	44(75)	1		1		1		1	
=10	15(25)	1.7^c^	1.1-2.8	2.2^b^	1.3-3.8	2.6^a^	1.0-7.1	2.5^a^	1.1-5.4
**Dyspnea**									
Absent	29(49)	1							
Present	30(51)	1.1	0.7-1.9						
**BMI(Kg/m^2^)**									
Nonwasted	24(41)	1		1		1		1	
Wasted	35(59)	1.4^c^	0.8-2.3	1.0	0.6-1.7	0.7	0.3-1.3	2.0	0.8-3.3
**LMI(Kg/m2)**									
Nonwasted	39(66)	1							
Wasted	20(34)	1.1	0.6-1.7						
**Clinical TB-**									
**severity**									
Mild	20(34)	1		1		1		1	
Moderate/severe	39(66)	1.5^c^	0.9-2.7	2.0^a^	1.0-3.5	2.5^a^	1.1-5.5	1.3	0.5-3.5

a p-value <0.05

b p-value<0.01

c p-value<0.25

Moderate/severe TB, BIA body wasting, male sex, dyspnea, and chest pain are associated with TC-hypocholesterolemia at univariate. At multivariate no factors were associated with TC-hypocholesterolemia (Table 4).

**Table 4:** Factors associated with TC-hypocholesterolemia among adult TB patients at diagnosis

Characteristic						Stratified models			
	
						HIV+(n=29)		HIV-(n=30)	
		Univariate		Overall model					
Overall(N)=59									
	N (%)	PR	95%CI	PR	95%CI	PR	95%CI	PR	95%CI
**Gender**									
Female	31(52)	1		1		1		1	
Males	28(48)	2.0^a^	1.1-3.1	2.0	0.8-3.7	4.0	0.8-19.5	1.6	0.5-4.5
**Age**									
=30	43(73)	1							
>30	16(27)	1.0	0.4-1.3						
**CD4cells**									
=200	20(34)	1							
>200	39(66)	1.2	0.7-2.1						
**HIV-serostatus**									
Negative	30(51)	1							
Positive	29(49)	1.0	0.5-1.4						
**Chest pain**									
Absent	21(36)	1		1		1		1	
Present	38(64)	2.0^c^	1.0-2.8	1.2	0.5-2.9	4.4	0.7-30.3	0.8	0.3-2.7
**Hemoptysis**									
Absent	49(83)	1							
Present	10(17)	1.1	0.6-2.0						
**Anemia, Hb**									
**g/dl**									
>10	44(75)	1							
= 10	15(25)	1.3	0.8-2.3						
**Dyspnea**									
Absent	29(49)	1		1		1		1	
Present	30(51)	2.0^c^	1.0-3.0	1.3	0.7-2.4	0.6	0.2-2.4	2.4	0.9-6.3
**BMI(Kg/m^2^)**									
Non-wasted	24(41)	1							
Wasted	35(59)	1.3	0.8-2.1						
**FFMI(Kg/m^2^)**									
Non-wasted	39(66)	1		1		1		1	
Wasted	20(34)	2.0^c^	0.9-2.6	1.0	0.5-2.1	0.4	0.1-1.7	2.0	0.5-4.9
**Clinical TB-severity**									
Mild	20(34)	1		1		1		1	
Moderate/severe	39(66)	2.0^a^	1.0-3.2	1.3	0.6-3.0	0.6	0.1-3.8	2.0	0.7-5.3

a p-value <0.05

b p-value<0.01

c p-value<0.25

**Figure 2:**
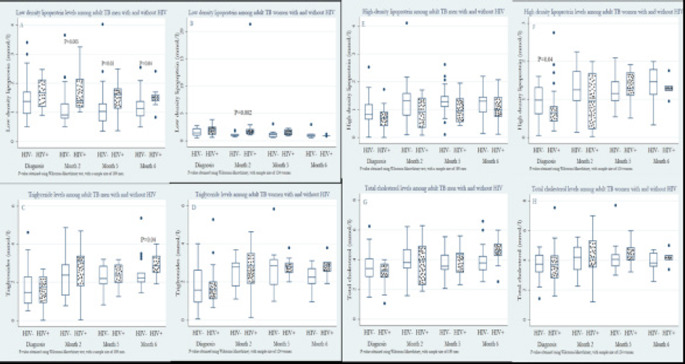
Box plots showing Lipid profiles among HIV-negative (HIV-) and HIV-positive (HIV+) men and women with TB(Tuberculosis) at varying times of anti-TB treatment. Panels A B, C D, G H showed Low density lipoprotein (LDL), Triglycerides (TG) and Total cholesterol (TC) among adult TB men and women with and without HIV. The recovery pattern of cholesterol (LDL, TG, TC) among men and women is similar among HIV- and HIV+ participants with TB. Higher cholesterol among coinfected participants at varying times is due to Antiretroviral (ARVs) treatment. Panels E, D showed High density lipoprotein (HDL) among TB men and women. In the presence of TB, HIV destroys HDL at diagnosis and presents a similar recovery pattern among HIV- and HIV+ participants among men and women. Significantly lower HDL in coinfections at diagnosis was due to TB which replicates HIV virus, a mechanism that requires more cholesterol for assembly of the viral envelope

Cholesterol recovery pattern was similar regardless of sex and HIV-serostatus. The recovery pattern was significantly lower in HIV-seronegative than HIV-seropositive participants with TB regardless of sex (Figure 2 AB, CD, GH). Regardless of sex, HDL recovery pattern was similar though was significantly lower in coinfection at diagnosis(Figure 2F).

**Figure 3:**
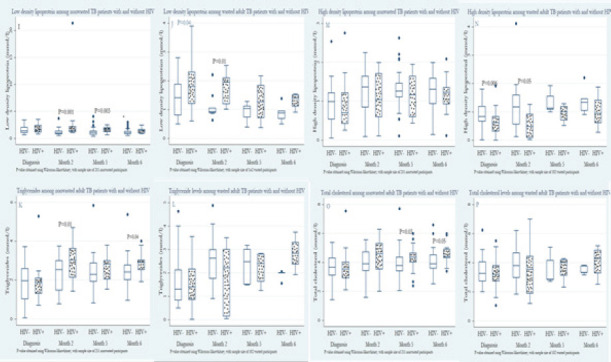
Box plots showing Lipid profiles among HIV-negative (HIV-) and HIV-positive (HIV+) nonwasted and wasted participants with TB(Tuberculosis) at varying times of anti-TB treatment. Panels (IJ, KL, OP) showed Low density lipoprotein (LDL), Triglycerides (TG) and Total cholesterol (TC) among adult TB patients with nonwasting and wasting with and without HIV. The cholesterol recovery among wasted and nonwasted participants was similar among HIV- and HIV+ participants with TB. The lower LDL among the wasted in TB compared with coinfection at diagnosis is attributed to very severe TB at the time of presentation. Panels (MN) showed High density lipoprotein (HDL) among adult TB patients with wasting and nonwasting with and without HIV. HDL recovery pattern at varying times among wasted and nonwasted participants was similar. At diagnosis and in the face of TB among the wasted, HIV had a more detrimental effect on HDL

Regardless of wasting status and HIV-serostatus, cholesterol recovery was similar (Figure 3 IJ, KL, OP). LDL was significantly lower among HIV-seronegative than HIV-seropositive participants at diagnosis among wasted participants(Figure 3J). Regardless of wasting status and HIV-serostatus, HDL trends were similar(Figure 3 MN). Among the wasted, HDL was significantly lower at diagnosis among coinfected participants(Figure 3N).

## Discussion

In this study, we hypothesized that TB severity among coinfected and TB patients was associated with hypocholesterolemia. Regardless of HIV-serostatus and sex, presence of TB posed a more detrimental effect on cholesterol with no effect on TG at diagnosis. Anemia, CD4>200cells and clinical TB-severity were associated with HDL-hypocholesterolemia. Male sex, clinical TB-severity, dyspnea, chest pain and body wasting were independently associated with TC-hypocholesterolemia. The recovery pattern of cholesterol was similar regardless of sex and wasting status.

We affirm that TB distorts cholesterol but not HIV regardless of sex. Similar findings were reported suggesting that inflammatory-immune activation stimulates release of macrophages and cytokines which interact with metabolic pathways leading to hypocholesterolemia^29^. However, presence of TB showed no effect on TG regardless of sex and HIV-status. Other studies have revealed TB detrimental effects on TG whereas others reported no effect^4^, ^30^.

Anemia in TB signified HDL-hypocholesterolemia regardless of HIV-status. Similar findings were reported among anemic patients though without TB^31^. Hypocholesterolemia is due to erythropoietin released by the kidney hence increasing erythropoiesis, a process requiring cholesterol^32^. Bidirectionally, inflammatory immune activation stimulates cytokines and interleukins that destroy cholesterol and convert iron into ferritin causing anemia^33^, ^34^.

Among coinfected participants, CD4>200cells indicated normal HDL with a strong immune system. Although poorly understood, other studies have reported similar findings suggesting that HIV viral gene and TB activate the immune system^35^. Proinflammatory cytokines from macrophages attack mycobacterium glycolipids and proteins activating CD4 recruitment which potentiate cytotoxic action^36^.

Finally, moderate/severe TB was suggestive of diminished HDL levels. Our findings are similar with previous studies where surrogates of clinical severity including sputum positivity and delayed sputum conversion were found to correlate with hypocholesterolemia^18^.

TB profoundly distorted cholesterol and remained lower at various times regardless of sex and wasting status. Our findings were similar with previous studies that showed higher cholesterol in coinfection due to increased fat redistribution after antiretroviral therapy initiation^37^. In the face of TB, HIV destroyed HDL and remained lower at various times. TB replicates HIV-virus, a process requiring more cholesterol for assembly of the viral envelope hence its decrease in HIV^38^.

The strength of our study is that we obtained lipid profiles from TB patients at various time points which discerns a wider scope of variation in lipid profile recovery. Nevertheless, findings may vary if similar participants were followed throughout treatment. We controlled for sex, wasting status and HIV-status to limit lipid profile differences.

### Study limitations

Our findings should be interpretated with caution as nutritional status was assessed using anthropometry and BIA. Anthropometry is associated with observer bias which was minimized by taking anthropometric values in duplicate. BIA was determined using equations that were cross-validated elsewhere but were used in African settings with meaningful results^39^. We lacked information on individual 24-hour dietary intake to demonstrate effects of diet on cholesterol overtime. Since our study was cross-sectional, we did not assess for causality. Nonetheless we enrolled participants at varying times to mimic a cohort study design. Data missingness on certain variables was observed leading to bias in estimates, however 15% data missingness was considered during sample size calculation^24^.

## Conclusion and recommendations

The present study revealed that, TB destroys cholesterol regardless of HIV-status and sex. Anemia, CD4>200cells and TB-severity were associated with HDL-hypocholesterolemia. Male sex, TB-severity, dyspnea, chest pain and body wasting were independently associated with TC-hypocholesterolemia. Variations in cholesterol recovery were observed at different times of treatment. Further research employing longitudinal/cohort study designs is ideal in elucidation of variations in cholesterol based on sex, wasting status and HIV-status. Routine lipid profiling in clinical practice is encouraged in the management of TB. Furthermore, randomized controlled trial should be conducted to demonstrate the recovery effect of a cholesterol pill on TB and body wasting^40^.

## References

[1] McQuaid CF, Vassall A, Cohen T, Fiekert K, White RG (2021). The impact of COVID-19 on TB: A review of the data. Int J Tuberc Lung Dis.

[2] Uganda population (2020). Tuberculosis profile. Estimates of TB burden are produced by WHO in consultation with countries. Generated 2022-01-31. Version 2.1.

[3] Annabel B, Anna D, Hannah M (2019). Global tuberculosis report.

[4] Akpovi L, Gbaguidi L, Anago E, Affolabi D, Dougnon TV, Faihun F (2013). Tuberculosis treatment raises total cholesterol level and restores high density lipoprotein cholesterol (HDL-C) in patients with pulmonary tuberculosis. Afr J Biotechno.

[5] Vander LE, Meeks K, Beune E, Amade-Graft A, Addo J, Owusu-Dabo E (2019). Dyslipidaemia among Ghanaian migrants in three European countries and their compatriots in rural and urban Ghana: the RODAM study. Atherosclerosis.

[6] Noubiap JJ, Bigna JJ, Nansseu JR, Nyaga UF, Balti EV, Echouffo-Tcheugui JB (2018). Prevalence of dyslipidaemia among adults in Africa: a systematic review and meta-analysis. Lancet Glob Health.

[7] Njelekela MA, Negishi H, Nara Y, Sato T, Tomohiro M, Kuga S (2002). Obesity and lipid profiles in middle aged men and women in Tanzania. East Afr Med J.

[8] Crevel RV, Karyadi E, Netea MG, Verhoef H, Nelwan RHH, West CE (2002). Decreased Plasma Leptin Concentrations in Tuberculosis Patients Are Associated with Wasting and Inflammation. J Clin Endocrinol Metab.

[9] Rajopadhye SH, Mukherjee SR, Chowdhary AS, Dandekar SP (2017). Oxidative Stress Markers in Tuberculosis and HIV/TB Co-Infection. J Clin Diagn Res.

[10] Weiner J, Parida SK, Maertzdorf J, Black GF, Repsilber D, Telaar A (2012). Biomarkers of inflammation, immunosuppression and stress with active disease are revealed by metabolomic profiling of tuberculosis patients. PLoS One.

[11] Tumusiime C, Katamba A, Nakiyingi L, Kalyango J Treatment Success and associated factors among patients with Pulmonary Tuberculosis attending Kampala Capital City Authority health facilities: A retrospective cohort study. MedRxiv.

[12] Warring F C, Sutramongkole U (1970). Nonculturable acid-fast forms in the sputum of patients with tuberculosis and chronic pulmonary disease. Am Rev Respir Dis.

[13] How SH, Kuan YC, Ng TH, Razali MR, Fauzi AR (2014). Monitoring treatment response in sputum smear positive pulmonary tuberculosis patients: comparison of weight gain, sputum conversion and chest radiograph. Malays J Pathol.

[14] Monika A, Ashisha B, Vinay B, Sarjana D (2016). Comparative study of GeneXpert with ZN stain and Culture in samples of suspected Pulmonary Tuberculosis. J Clin Diagn Res.

[15] MoH(Uganda) (2017). Uganda National Tuberculosis and Leprosy Control Programme. Manual for Management and Control of Tuberculosis and Leprosy.

[16] World Health Organisation (2025). Consolidated Guidelines Operational Handbooks Training Catalogue Research and Innovation. WHO TB Knowledge sharing platform.

[17] Jameel BF, Sultan QM, Naser AA (2012). Assessment of Body Mass Indexand Nutritional Status in Pulmonary Tuberculosis patients. Iraqi J Comm Med.

[18] Taparia P, Yadav D, Koolwal S, Mishra S (2015). Study of lipid profile in pulmonary tuberculosis patients and relapse cases in relation with disease severity-A pilot study. IJSAR.

[19] MoH(Uganda) (2005). Uganda National Policy Guidelines for HIV counselling and testing.

[20] Kyle UG, Piccoli A, Pichard C (2003). Body composition measurements: interpretation finally made easy for clinical use. Clin Nutr Metab Care.

[21] Wejse C, Gustafson P, Nielsen J, Gomes VF, Aaby P, Andersen PL (2008). TBscore:Signs and symptoms from tuberculosis patients in a low-resource setting have predictive value and may be used to assess clinical course. Scand J Infect Dis.

[22] Michael B, Clearfield DO (2003). The National Cholesterol Education Program Adult Treatment Panel III guidelines. JAOA.

[23] Mukisa J, Kawooya I, Nangendo J, Nalutaaya A, Nyamwiza J, Sam A (2018). Male gender and duration of anti-tuberculosis treatment are associated with hypocholesterolemia in adult pulmonary tuberculosis patients in Kampala, Uganda. Afri Health Sci.

[24] Sumeyra S, Haydar K, Nuri D (2018). The effects of sample size and missing data rates on generalizability coefficients. EJER.

[25] Lin DY, Wei LJ (1989). The robust inference for the Cox proportional hazards model. JAm Stat Assoc.

[26] Lee J, Chia KS (1993). Estimation of prevalence rate ratios for cross sectional data: an example in occupational epidemiology. Br J Ind Med.

[27] Coutinho LMS, Marcia Scazufca M, Menezes PR (2008). Methods for estimating prevalence ratios in crosssectional studies. Rev Saúde Pública.

[28] Heinze G, Wallisch C, Dunkler D (2018). Variable selection – A review and recommendations for the practicing statistician. Biom-J.

[29] Parchwani DSP, Patel SD (2011). Total antioxidant status and lipid peroxides in patients with pulmonary tuberculosis. Nati J Community-med.

[30] Sushilendu V, Kumar N, Kumar U (2019). Study of Lipid profile in pulmonary TB cases: pre and post anti-tuberculosis treatment. JMSCR.

[31] Beeravelli S, Kinathankaraiyan N, Manickam R (2023). A Cross-Sectional Study of Lipid Profile in Anemic Patients in a Tertiary Care Center. Assam J Intern-Med.

[32] Lu Z, Huang L, Li Y, Xu Y, Zhang R, Zhou Q (2022). Fine-Tuning of Cholesterol Homeostasis Controls Erythroid Differentiation. Adv Sci.

[33] Means R T (2003). Recent developments in the anemia of chronic disease. Curr Hematol Rep.

[34] Nemeth E, Rivera S, Gabayan V (2004). IL-6 mediates hypoferremia of inflammation by inducing the synthesis of the iron-regulatory hormone hepcidin. J Clin invest.

[35] Toossi Z (2003). Virological and immunological impact of tuberculosis on human immunodeficiency virus type 1 disease. J Infect Dis.

[36] Dabrowski MP, Peel WE, Thomson AER (1980). “Plasma membrane cholesterol regulates human lymphocyte cytotoxic function”. Eur J Immunol.

[37] Oduola T, Akinbolade AA, Oladokun LO, Adeosun OG, Bello IS, Ipadeola TI (2009). Lipid Profiles in People Living with HIV/AIDS on ARV Therapy in an Urban Area of Osun State, Nigeria. WJMS.

[38] Riddler SA SE, Cole SR, Li R, Chmiel JS, Dobs A (2003). Impact of HIV infection and HAART on serum lipids in men. JAMA.

[39] Kotler D, Burastero S, Wang J, Pierson RN (1996). Prediction of body cell mass, fat-free mass, and total body water with bioelectrical impedance analysis: effects of race, sex, and disease. Am J Clin Nutr.

[40] Perez-Guzman C, Vargas MH, Quinonen F, Bazavilvazo N, Aguilar A (2005). A cholesterol-rich Diet Accelerates Bacteriologic Sterilization in Pulmonary Tuberculosis. Chest.

